# Deficiency of MicroRNA-23-27-24 Clusters Exhibits the Impairment of Myelination in the Central Nervous System

**DOI:** 10.1155/2023/8938674

**Published:** 2023-03-22

**Authors:** Yuji Tsuchikawa, Naosuke Kamei, Yohei Sanada, Toshio Nakamae, Takahiro Harada, Kazunori Imaizumi, Takayuki Akimoto, Shigeru Miyaki, Nobuo Adachi

**Affiliations:** ^1^Department of Orthopaedic Surgery, Graduate School of Biomedical and Health Sciences, Hiroshima University, Hiroshima 7348551, Japan; ^2^Medical Center for Translational and Clinical Research, Hiroshima University Hospital, Hiroshima 7348551, Japan; ^3^Department of Biochemistry, Graduate School of Biomedical and Health Sciences, Hiroshima University, Hiroshima 7348551, Japan; ^4^Faculty of Sport Sciences, Waseda University, Tokorozawa 3591192, Japan

## Abstract

Several microRNAs (miRNAs), including miR-23 and miR-27a have been reportedly involved in regulating myelination in the central nervous system. Although miR-23 and miR-27a form clusters *in vivo* and the clustered miRNAs are known to perform complementary functions, the role of these miRNA clusters in myelination has not been studied. To investigate the role of miR-23-27-24 clusters in myelination, we generated miR-23-27-24 cluster knockout mice and evaluated myelination in the brain and spinal cord. Our results showed that 10-week-old knockout mice had reduced motor function in the hanging wire test compared to the wild-type mice. At 4 weeks, 10 weeks, and 12 months of age, knockout mice showed reduced myelination compared to wild-type mice. The expression levels of myelin basic protein and myelin proteolipid protein were also significantly lower in the knockout mice compared to the wild-type mice. Although differentiation of oligodendrocyte progenitor cells to oligodendrocytes was not inhibited in the knockout mice, the percentage of oligodendrocytes expressing myelin basic protein was significantly lower in 4-week-old knockout mice than that in wild-type mice. Proteome analysis and western blotting showed increased expression of leucine-zipper-like transcription regulator 1 (LZTR1) and decreased expression of R-RAS and phosphorylated extracellular signal-regulated kinase 1/2 (pERK1/2) in the knockout mice. In summary, loss of miR-23-27-24 clusters reduces myelination and compromises motor functions in mice. Further, LZTR1, which regulates R-RAS upstream of the ERK1/2 pathway, a signal that promotes myelination, has been identified as a novel target of the miR-23-27-24 cluster in this study.

## 1. Introduction

In the central nervous system (CNS), myelin, produced by oligodendrocytes, supports axonal metabolism and allows for rapid transmission of action potentials along axons [1–3]. In demyelinating conditions such as multiple sclerosis or spinal cord injury, myelin is lost and axons degenerate, resulting in permanent loss of function. Remyelination in these demyelinating conditions remains a challenge. Developmental myelination and remyelination have the common goal of attaching myelin sheaths to axons [4]. In addition, there is a common mechanism underlying developmental myelination and remyelination, and remyelination is considered as “rerunning” of myelination [4]. Therefore, understanding the mechanism underlying myelination will help in developing novel strategies for remyelination in various disease conditions. Normal myelination is a multistep process in which oligodendrocyte differentiation and initiation of myelination are tightly regulated [5]. Previous studies have shown that several genes, transcription factors, and pathways are involved in oligodendrocyte progenitor cell (OPC) proliferation and oligodendrocyte differentiation, maturation, and myelination [6–8]. Furthermore, recent studies have shown that microribonucleic acids (miRNAs) play important roles in regulating myelination [9–15].

miRNAs are small (~22 nucleotides long) noncoding RNAs that act as master regulators of gene expression [16]. Deletion of Dicer1, a processing enzyme essential for miRNA maturation, markedly impairs oligodendrocyte differentiation and myelination [10, 11], highlighting the important role of miRNAs in regulating CNS myelination. Herein, we focused on the role of the miR-23-27-24 cluster, which is highly expressed in oligodendrocytes [17]. There are two paralogous miR-23-27-24 clusters, miR-23a-27a-24-2 (miR-23a cluster) and miR-23b-27b-24-1 (miR-23b cluster), located on chromosomes 8 and 13, respectively, in mice, and on chromosomes 19 and 9, respectively, in humans [18]. Recent studies have reported that miR-23a, miR-23b, and miR-27a are individually involved in regulating myelination *in vitro* model [9, 14]. In addition, transgenic mice overexpressing miR-23a promote myelination [9]. However, the functions of these miRNAs in a cluster unit have not yet been elucidated using *in vivo* model. Clustered miRNAs regulate common molecular pathways in a complementary manner [13]. Therefore, deletion of one miRNA in the cluster may not induce a clear change in the target gene expression because other miRNAs will compensate for its function. Since a single miRNA can target multiple genes and miRNA clusters contain multiple miRNAs, it is important to understand the regulation of miRNAs in a cluster unit and their various biological functions [19]. To understand the role of the miR-23-27-24 cluster in myelination in vivo, we generated miR-23a/b cluster knockout (miR-23 KO) mice. Effect of miR-23-27-24 cluster deletion on motor functions was evaluated using standard behavioral assays. Evaluation of white matter hypoplasia and oligodendrocyte differentiation in the spinal cord and brain was performed by histological methods. To assess the effect of miR-23-27-24 cluster deletion on myelination, we performed electron microscopy on spinal cord sections. Finally, to identify the targets of miR-23-27-24 cluster miRNAs, global proteome analysis and western blotting were performed.

## 2. Materials and Methods

All animal care and handling procedures were performed according to the Guidelines for Proper Conduct of Animal Experiments by the Science Council of Japan and approved by the Committee of Research Facilities for Laboratory Animal Sciences, Graduate School of Biomedical Sciences, Hiroshima University (Approval No. A18-134).

### 2.1. Animals

All mice were housed individually and were maintained on a 12 h light/12 h dark cycle at room temperature (22 ± 2°C) with 40-60% humidity. Both males and females were used in this experiment. miR-23a/b-cluster-double-floxed mice [18] and CAG-cre mice (B6.Cg-Tg(CAG-cre) CZ-MO2Osb, Riken BRC, Ibaraki, Japan) [20] were previously described. The global miR-23 KO mice were generated by crossing miR-23a/b-cluster-double-floxed mice with CAG-cre mice. Mice were genotyped using tail tip genomic DNA as described [18]. The miR-23 KO mice were identified with PCR using the specific primers: 5′-GCA TTA CCG GTC GAT GCA ACG AGT GTA GAG-3′ and 5′-GAG TGA ACG AAC CTG GTC GAA ATC AGT GCG-3′.

### 2.2. Real-Time PCR

The expression of the miR-23-27-24 cluster miRNAs (miR-23a, miR-23b, miR-27a, miR-27b, miR-24) in 4-week-, 10-week-, and 12-month-old wild-type and miR-23-27-24 cluster knockout mice was evaluated by real-time polymerase chain reaction (PCR). Briefly, mice were anesthetized with isoflurane and transcardially perfused with 20 ml of sterile RNase-free phosphate-buffered saline (PBS). The spinal cord and brain were collected from four mice of each genotype at 4 weeks, 10 weeks, and 12 months of age. Total RNA was extracted from these tissues using ISOGEN (Nippon Gene, Tokyo, Japan) according to the manufacturer's instructions. To synthesize first-strand complementary deoxyribonucleic acid (DNA), total RNA was reverse-transcribed using the TaqMan™ MicroRNA Reverse Transcription Kit (Thermo Fisher Scientific, Waltham, MA, USA). TaqMan™ MicroRNA Assays (Thermo Fisher Scientific) for miR-23a (000399), miR-23b (000400), miR-27a (000408), miR-27b (000409), and miR-24 (000402) were used to analyze miRNA expression. Real-time PCR was performed using the Applied Biosystems StepOne™ Real-time PCR System (Thermo Fisher Scientific) in 96-well plates. The expression of each miRNA was normalized to the expression level of U6 small nuclear RNA (001973).

### 2.3. Behavioral Assessment

Behavioral assessment was performed on 4-week-, 10-week-, and 12-month-old wild-type and miR-23 KO mice. The following motor function tests were conducted as described in previous reports: open field test [21], hanging wire test [22], and balance beam test [12]. In the open field test, a video tracking system (SMART 3.0, Panlab, Barcelona, Spain) was used to assess the behavior. The mouse was placed in the center of the open field box, and the distance traveled in 3 min was measured. For each mouse, the measurement was performed three times, and the average value was calculated. The hanging wire test was used to assess global subacute muscle function and coordination, as previously described [23]. In this test, a 2 mm thick metallic wire was secured to two vertical stands. The mouse was placed at the center of the wire, and the time until the mouse dropped off was measured. The test was performed three times for each mouse, and the average value was calculated. The balance beam test was performed to assess motor coordination and balance. A 10 mm wide and 50 cm long beam was used in this study. The time taken to cross the beam was recorded for each mouse. A brightly illuminated start platform and a nonilluminated escape box were situated at each end of the beam. The measurements were performed three times for each mouse, and the minimum time was recorded.

All experiments were performed during the dark cycle since the animals are more active during this period. The experimenters were blinded to the genotype of each mouse during behavioral tests and subsequent analyses.

### 2.4. Luxol Fast Blue Staining

White matter in the brain and spinal cord of 4-week-, 10-week-, and 12-week-old wild-type and miR-23-27-24 cluster knockout mice was evaluated by Luxol Fast Blue staining. The mice were transcardially perfused with 20 ml of PBS followed by 40 ml of freshly prepared 4% paraformaldehyde (PFA). Whole brain and the spinal cord at the 9th thoracic level were collected and fixed in PFA for 8 h and then incubated in sucrose solutions of increasing concentration (15% and 30%) at 4°C. After, the tissue was submerged in OCT compound, frozen, and stored at -80°C. Fixed tissues were sectioned to a thickness of 15 *μ*m using a cryostat. Frozen sections were collected on slides, thawed, rehydrated in PBS, incubated with Luxol Fast Blue Solution (LBC-2-IFU, ScyTek Laboratories, Logan, UT) in a sealed container for 8 h at 60°C, washed with distilled water, and placed in 95% alcohol for 10 min. Sections were then incubated in a 0.05% lithium carbonate aqueous solution for 10 s and in a 70% alcohol solution for 20 s. The above two steps were repeated until the gray and white matter was clearly observable under the microscope. To determine the thickness of the corpus callosum, 20 continuous coronal sections of the splenium of the corpus callosum were obtained. The thickness of the corpus callosum in these 20 sections was measured, and the maximum value was used for analysis. In the spinal cord transverse sections, the area of the entire spinal cord and the area of the white matter were measured. The ratio of the white matter area to the total area of the spinal cord was calculated as the ratio of white matter. Three consecutive sections were used to calculate the ratio of white matter, and the average values of these sections were used for the analysis. Five mice from each genotype were examined. ImageJ (National Institutes of Health, Bethesda, MD, RRID:SCR_003070) was used to measure the length and area.

### 2.5. Electron Microscopy

Electron microscopy was performed to evaluate myelination in the spinal cord of 4-week-, 10-week-, and 12-month-old wild-type and miR-23-27-24 cluster knockout mice. Mice were anesthetized with isoflurane and perfused briefly with PBS followed by 2% glutaraldehyde in 0.1 M cacodylate (pH 7.2). The spinal cord at 9th thoracic level was removed and fixed in fresh fixative overnight at 4°C. Tissues were rinsed in PBS, postfixed in 1.5% OsO_4_ in PBS for 2 h, dehydrated in graded ethanol solutions, infiltrated with propylene oxide, and embedded in Epon. Semithin sections were stained with toluidine blue, and 70-80 nm thin sections were stained with 2% uranyl acetate and lead citrate. The ventrolateral side of the white matter of the spinal cord was the area of interest. The G-ratio of axons in the area of interest was obtained as the ratio of the diameter of an axon to the diameter of the axon plus the associated myelin sheath. Approximately 150-200 axons per mouse and three mice per group were analyzed. Digitized and calibrated images were analyzed using ImageJ.

### 2.6. Immunohistochemistry

The corpus callosum coronal sections and the spinal cord transverse sections were immunostained to evaluate oligodendrocytes. These sections were permeabilized with 0.3% Triton X-100, and nonspecific binding sites were blocked with serum matching the species of the secondary antibody. Where anti-mouse IgG monoclonal antibodies were used, sections were additionally incubated with mouse IgG blocking reagent (Vector Laboratories, Burlingame, CA). The sections were then stained with the following primary antibodies: rabbit anti-Olig-2 (1 : 500, AB9610, Sigma-Aldrich, St. Louis, MO, RRID:AB_570666), goat anti-platelet-derived growth factor receptor alpha (PDGFR*α*, 1 : 200, AF1062, R&D Systems, Minneapolis, MN, RRID:AB_2236897), mouse anti-CC1 (1 : 100, OP80, Sigma-Aldrich, RRID:AB_2057371), and rat anti-myelin basic protein (MBP, 1 : 250, ab7349, Abcam, Cambridge, UK, RRID:AB_305869). The following secondary antibodies (1 : 500, Thermo Fisher Scientific) were used: Alexa Fluor 350-conjugated donkey anti-goat, Alexa Fluor 488-conjugated donkey anti-rabbit, Alexa Fluor 594-conjugated donkey anti-mouse, Alexa Fluor 594-conjugated goat anti-rat, and Alexa Fluor 594-conjugated goat anti-rabbit. Immunostained sections were observed under a fluorescence microscope (BZ9000; Keyence, Osaka, Japan).

### 2.7. Comprehensive Proteome Analysis

Quantitative analysis of more than 7000 proteins was performed by the data-independent acquisition (DIA) proteome analysis using Q Exactive™ HF-X (Thermo Fisher Scientific) as described previously [24]. DIA proteome analysis was performed on spinal cord white matter tissues from 10-week-old wild-type and KO mice. Mice were anesthetized with isoflurane and briefly perfused with PBS. Spinal cord white matter tissues were collected and immediately frozen at -80°C. Frozen samples were sent to Kazusa Genome Technologies (Kisarazu, Japan) for further analysis.

### 2.8. Western Blotting Analysis

Spinal cord white matter tissues from 4-week- and 10-week-old wild-type and KO mice were homogenized in T-PER (Thermo Fisher Scientific) containing a protease inhibitor cocktail Set I (Fujifilm Wako Pure Chemical, Osaka, Japan) and centrifuged at 10,000 × *g* for 5 min. The supernatant protein was separated by 10% SDS polyacrylamide gel electrophoresis and electrophoretically transferred to polyvinylidene difluoride membranes. Membranes were blocked for 30 min at room temperature with 5% skimmed milk (Fujifilm Wako Pure Chemical) and then incubated with the following primary antibodies: rabbit anti-MBP (1 : 1000, ab40390 Abcam, RRID:AB_1141521), rabbit anti-myelin proteolipid (PLP, 1 : 1000, ab105784, Abcam, RRID:AB_10973392), rabbit anti-phosphatase and tensin homolog deleted on chromosome 10 (PTEN, 1 : 500, ab32199, Abcam, RRID:AB_777535), rabbit anti-lamin B1 (1 : 1000, ab16048 Abcam, RRID:AB_10107828), rabbit anti-LZTR1 (1 : 1000, ab106655, Abcam), rabbit anti-R-RAS (1 : 500, ab154962, Abcam), rabbit anti-Akt (1 : 1000, #9272 Cell Signaling Technology, Danvers, MA, RRID:AB_329827), rabbit anti-phospho-Akt (1 : 2000, p-Akt, #4060 Cell Signaling Technology, RRID:AB_2315049), rabbit anti-Erk1/2 (1 : 1000, #9102 Cell Signaling Technology, RRID:AB_330744), and rabbit anti-phospho-Erk1/2 (p-Erk1/2, 1 : 1000, #9101, Cell Signaling Technology, RRID:AB_331646), followed by the secondary horseradish peroxidase- (HRP-) conjugated anti-rabbit IgG antibody. In addition, peroxide-conjugated mouse anti-glyceraldehyde-3-phosphate dehydrogenase (GAPDH, 1 : 5000, 015-25473, Fujifilm Wako Pure Chemical) was used for GAPDH expression analysis. The blots were visualized using Amersham ECL Detection Reagents (Cytiva, Marlborough, MA). The expression of each protein was normalized to that of GAPDH.

### 2.9. Statistical Analysis

All measured values were expressed as mean ± standard deviation. Data were analyzed using JMP® 15 (SAS Institute Inc., Cary, NC, USA, RRID:SCR_014242). Student's unpaired *t*-test was used for comparing two groups. One-way analysis of variance (ANOVA) followed by Tukey's post hoc test was performed for multiple comparisons. *p* < 0.05 was considered statistically significant.

## 3. Results

### 3.1. Expression of the miR-23-27-24 Cluster in the CNS

**Figure 1:**
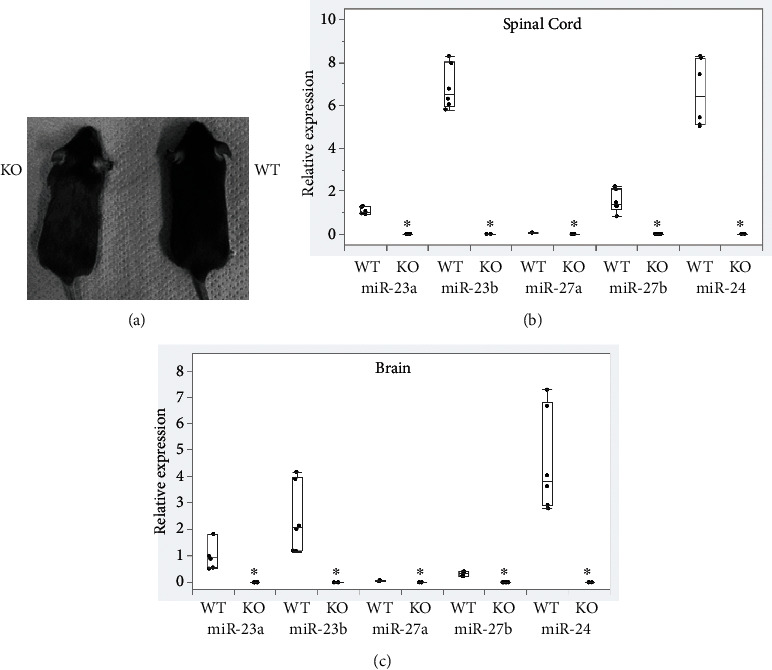
Characteristics of the miR-23-27-24 cluster knockout mice. Wild-type (WT) and miR-23-27-24 cluster knockout (KO). (a) Appearance of the 10-week-old WT and KO mice. (b) Expression levels of the miR-23-27-24 cluster miRNAs in the spinal cord of 10-week-old WT and KO mice, *n* = 4 per genotype. (c) Expression levels of the miR-23-27-24 cluster miRNAs in the brain of 10-week-old WT and KO mice, *n* = 4 per genotype. In the KO mice, the expression of all miRNAs was significantly lower in both the spinal cord and brain. ^∗^Significant difference compared to wild-type mice, *p* < 0.05.

KO mice were slightly smaller than wild-type mice but were born and grew without morphological abnormalities (Figure 1(a)). Compared to 10-week-old wild-type mice, the expression of all miRNAs in the miR-23-27-24 cluster was significantly lower in the KO mice, both in the spinal cord and brain (*p* < 0.001) (Figures 1(b) and 1(c) and Table 1).

**Figure 2:**
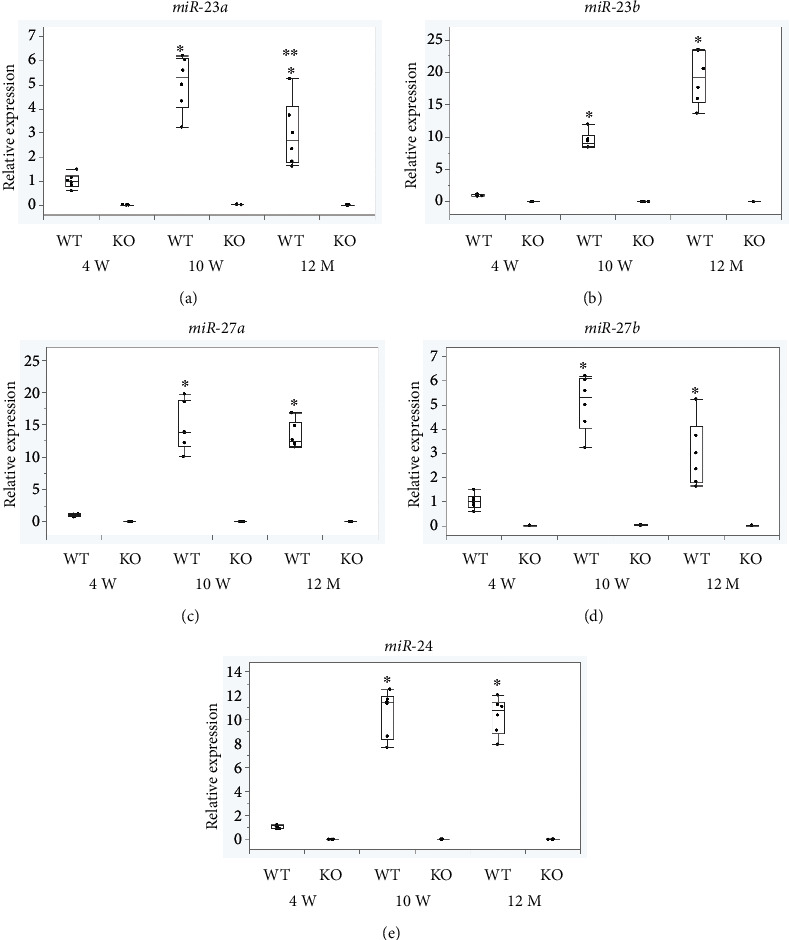
Expression levels of the miR-23-27-24 cluster miRNAs in the spinal cord of wild-type mice at different ages. (a) miR-23a, (b) miR-23b, (c) miR-27a, (d) miR-27b, and (e) miR-24, *n* = 4 per group. The expression of all miRNAs was significantly higher in 10-week-old mice compared to 4-week-old mice. miR-23a expression was lower in 12-month-old mice compared to 10-week-old mice. ^∗^Compared to 4-week-old. ^∗∗^Compared to 10-week-old, *p* < 0.05.

Next, we compared the expression of the miR-23-27-24 cluster in the spinal cord of mice at different ages (4 weeks, 10 weeks, and 12 months of age). For all miRNAs in the miR-23-27-24 cluster, the expression levels in 10- and 12-week-old wild-type mice were significantly higher than those in 4-week-old wild-type mice (*p* < 0.001) (Figure 2). The expression of miR-23a in 12-month-old wild-type mice was significantly lower than that in 10-week-old wild-type mice (*p* < 0.001) (Figure 2(a)), but there were no significant differences in the expression of other miRNAs (Figures 2(b)–2(e)). The expression of all miRNAs in KO mice was significantly lower than that in wild-type mice at all ages (Table 2).

### 3.2. Knockdown of the miR-23-27-24 Cluster Results in Decreased Motor Function

**Figure 3:**
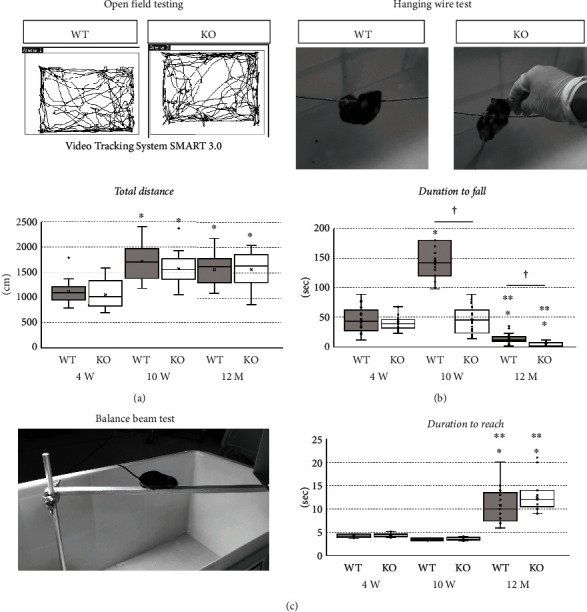
Behavioral assessment. Wild-type (WT) and miR-23-27-24 cluster knockout (KO), *n* = 8 per group. (a) Open field test. Total distance travelled by the mouse in 3 min was measured. In both WT and KO mice, the total distance travelled by the 10-week-old and 12-month-old mice was significantly more than the distance travelled by the 4-week-old mice. There was no significant difference between WT mice and KO mice at all ages. (b) Hanging wire test. The time until the mouse fell off from the wire was measured (duration to fall). In both WT and KO mice, the duration to fall was significantly shorter at 12 months of age than that at 4 or 10 weeks of age. In contrast, the duration to fall at 10 weeks of age was significantly longer than that at 4 weeks of age only in WT mice. The duration to fall in KO mice was significantly shorter than that in WT mice at 10 weeks of age. ^∗^Compared to 4-week-old. ^∗∗^Compared to 10-week-old. ^†^Comparison between WT and KO mice, *p* < 0.05. (c) Balance beam test. The time taken for the mouse to cross the beam was measured (duration to reach). The duration to reach was significantly longer at 12 months of age than that at 4 or 10 weeks of age. There was no significant difference between WT mice and KO mice at all ages. ^∗^Compared to 4-week-old. ^∗∗^Compared to 10-week-old, *p* < 0.05.

To evaluate the effect of miR-23-27-24 cluster deletion on mice behavior, we performed the open field test, hanging wire test, and balance beam test (Figure 3 and Table 3). One-way ANOVA showed significant differences between groups (*F*(5, 36) = 7.217, *p* < 0.001 in the open field test; *F*(5, 37) = 80.918, *p* < 0.001 in the hanging wire test; *F*(5, 37) = 47.405, *p* < 0.001 in the balance beam test). In the open field test, the total distance traveled by the 4-week-old mice was significantly lower than that travelled by the 10-week- (*p* = 0.002 in wild-type mice, *p* = 0.010 in KO mice) and 12-month-old mice (*p* = 0.033 in wild-type mice, *p* = 0.008 in KO mice) in both wild-type and KO groups (Figure 3(a)). In the hanging wire test, the latency to fall was significantly lower in 12-month-old mice compared to 4-week- (*p* = 0.004 in wild-type mice, *p* = 0.002 in KO mice) and 10-week-old mice (*p* < 0.001 in wild-type and KO mice) in both wild-type and KO groups (Figure 3(b)). In the balance beam test, the time taken by the 12-month-old mice to cross the beam was significantly longer compared to time taken by 4- and 10-week-old mice in both wild-type and KO groups (*p* < 0.001) (Figure 3(c)). Thus, growth-associated development and aging-associated decline in motor functions were observed in both wild-type and KO mice.

In the open field and balance beam tests, no significant differences were observed between the wild-type and KO mice at all ages (Figures 3(a) and 3(c)). However, in the hanging wire test, which requires general motor skills such as grip strength and balance function, the 10-week-old KO mice performed significantly worse than 10-week-old wild-type mice (*p* < 0.001) (Figure 3(b)). In addition, although the latency to fall in the hanging wire test was significantly higher in 10-week-old wild-type mice compared to that in 4-week-old wild-type mice (*p* < 0.001), there was no significant difference between 4- and 10-week-old KO mice (*p* = 0.914) (Figure 3(b)). These findings indicate that some motor functions are compromised in KO mice.

### 3.3. Knockdown of the miR-23-27-24 Cluster Leads to Hypomyelination in the CNS

**Figure 4:**
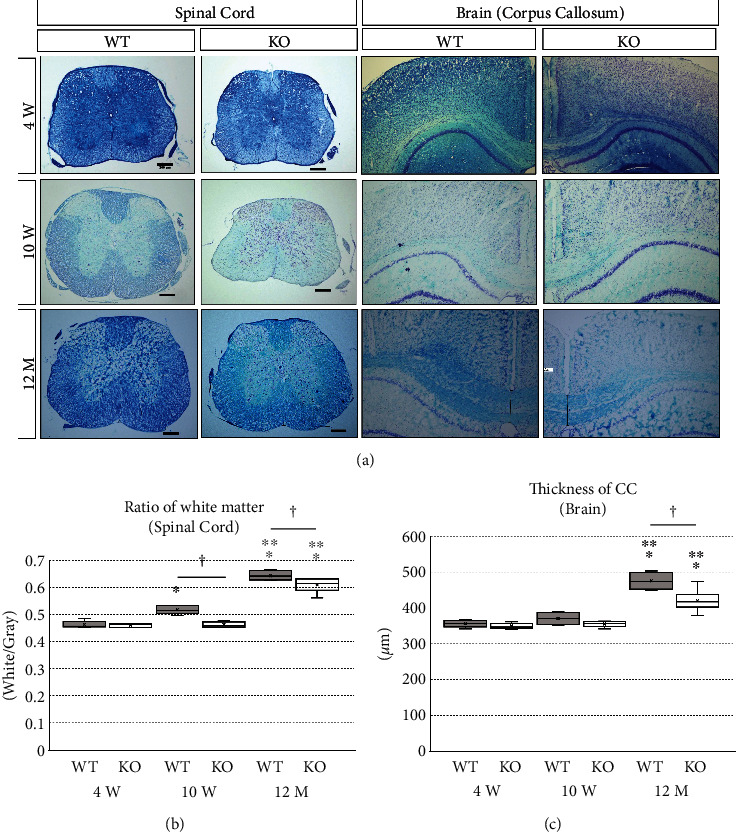
Luxol Fast Blue staining of the brain and spinal cord sections. Wild-type (WT) and miR-23-27-24 cluster knockout (KO), *n* = 5 per group. (a) Representative images of Luxol Fast Blue stained transverse sections of the thoracic spinal cord and coronal sections of the corpus callosum in WT and KO mice at 4 weeks, 10 weeks, and 12 months of age. Scale bars: 200 *μ*m. (b) The ratio of the white matter area to the total area of the spinal cord (ratio of white matter). ^∗^Compared to 4-week-old. ^∗∗^Compared to 10-week-old. ^†^Comparison between WT and KO mice, *p* < 0.05. (c) The thickness of the corpus callosum (CC). ^∗^Compared to 4-week-old. ^∗∗^Compared to 10-week-old. ^†^Comparison between WT and KO mice, *p* < 0.05.

To assess myelination, histological evaluation of the white matter was performed in 4-week-, 10-week-, and 12-month-old wild-type and KO mice. In the Luxol Fast Blue staining of transverse sections of the spinal cord (Figure 4(a)), the ratio of white matter was 0.46 ± 0.01 in 4-week-old wild-type mice, 0.46 ± 0.01 in 4-week-old KO mice, 0.52 ± 0.02 in 10-week-old wild-type mice, 0.46 ± 0.01 in 10-week-old KO mice, 0.64 ± 0.02 in 12-month-old wild-type mice, and 0.61 ± 0.03 in 12-month-old KO mice. One-way ANOVA showed significant differences between the groups (*F*(5, 24) = 106.711, *p* < 0.001). The ratio of white matter was significantly higher in 10-week-old wild-type mice compared to than in 4-week-old wild-type mice. In addition, the ratio of white matter in 12-month-old mice was significantly higher than that in 4- and 10-week-old mice of both genotypes (*p* < 0.001) (Figure 4(b)). Moreover, the ratio of white matter in KO mice was significantly lower than that in wild-type mice at 10 weeks (*p* < 0.001) and 12 months of age (*p* = 0.042) (Figure 4(b)). In the Luxol Fast Blue staining of coronal sections of the corpus callosum (Figure 4(a)), the thickness of corpus callosum was 356.00 ± 9.51 *μ*m in 4-week-old wild-type mice, 349.20 ± 7.95 in 4-week-old KO mice, 370.40 ± 17.13 in 10-week-old wild-type mice, 355.20 ± 8.04 in 10-week-old KO mice, 479.82 ± 23.27 in 12-month-old wild-type mice, and 420.50 ± 34.65 in 12-month-old KO mice. One-way ANOVA showed significant differences between the groups (*F*(5, 24) = 35.679, *p* < 0.001). The thickness of the corpus callosum in 12-month-old mice was significantly higher than that in 4- and 10-week-old mice of both genotypes (*p* < 0.001). Between wild-type and KO mice, the thickness of the corpus callosum was significantly lower in KO mice at 12 months of age (*p* < 0.001) (Figures 4(a) and 4(c)). These results indicate that there is no apparent difference in CNS white matter formation between wild-type and KO mice during the juvenile stage, but later, growth and aging lead to reduction in white matter formation in KO mice.

**Figure 5:**
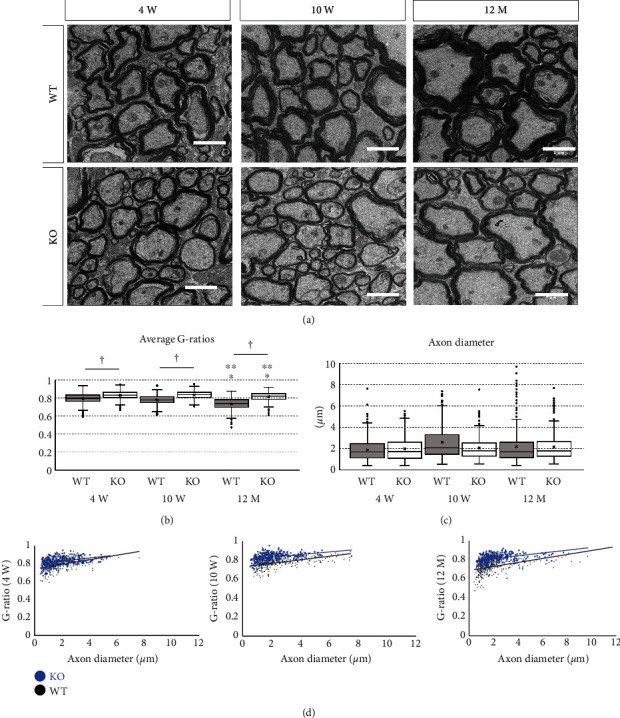
Evaluation of myelin sheath by electron microscopy. Wild-type (WT) and miR-23-27-24 cluster knockout (KO), 150-200 axons per mouse, *n* = 3 per group. (a) Electron micrograph of the transverse section of the spinal cord in WT and KO mice at 4 weeks, 10 weeks, and 12 months of age. Scale bars: 2 *μ*m. (b) The ratio of the diameter of the axon without myelin sheath to the diameter of the axon with myelin sheath (G-ratio) was used to evaluate the thickness of the myelin sheath. G-ratio in 12-month-old mice was significantly lower than that in 4- and 10-week-old WT and KO mice. G-ratio in 10-week-old WT mice was significantly lower than that in 4-week-old WT mice. G-ratio in KO mice was significantly higher than that in WT mice at all ages. ^∗^Compared to 4-week-old. ^∗∗^Compared to 10-week-old. ^†^Comparison between WT and KO mice, *p* < 0.05. (c) There was no significant difference in axon diameter between WT and KO mice or between mice of different ages. (d) Regardless of the axon diameter, the G-ratio in KO mice was higher than that in WT mice at all ages.

The thickness of the myelin sheath at different ages was evaluated by electron microscopy (Figure 5(a)). The thickness of the myelin sheath was assessed using the G-ratio, which is the ratio of the diameter of the axon excluding the myelin sheath to the diameter of the axon with the myelin sheath. The G-ratio was 0.79 ± 0.05 in 4-week-old wild-type mice, 0.83 ± 0.05 in 4-week-old KO mice, 0.78 ± 0.05 in 10-week-old wild-type mice, 0.83 ± 0.04 in 10-week-old KO mice, 0.73 ± 0.07 in 12-month-old wild-type mice, and 0.82 ± 0.05 in 12-month-old KO mice. One-way ANOVA showed significant differences between the groups (*F*(5, 2122) = 177.577, *p* < 0.001). In wild-type mice, the G-ratio in 10-week-old mice was significantly lower than that in 4-week-old mice (*p* = 0.001), and the G-ratio in 12-month-old mice was significantly lower than that in 4-week- (*p* < 0.001) and 10-week-old (*p* < 0.001) mice. On the other hand, in KO mice, the G-ratio in 12-month-old mice was significantly lower than that in 4-week- (*p* = 0.002) and 10-week-old (*p* < 0.001) mice, but there was no significant difference between 4-week- and 10-week-old mice (*p* = 0.957) (Figure 5(b)). Between wild-type and KO mice, the G-ratio in KO mice was significantly higher than that in wild-type mice at all ages (*p* < 0.001). In contrast, there was no significant difference in axon diameter between animals of different age or genotype (Figure 5(c)). The G-ratio in KO mice was higher than that in wild-type mice, regardless of the axon diameter at all ages (Figure 5(d)). These findings suggest that myelin sheath thickens with age and KO mice have a thinner myelin sheath compared to wild-type mice.

**Figure 6:**
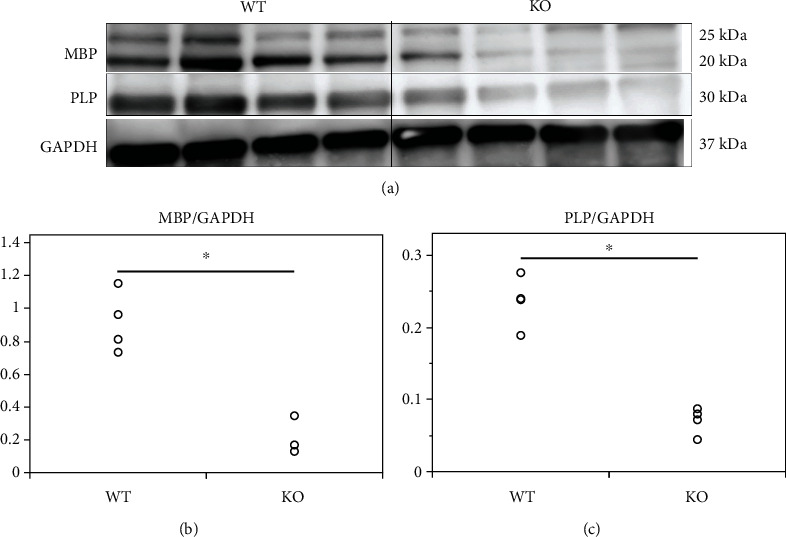
Expression of MBP and PLP in the spinal cord of 10-week-old mice. Wild-type (WT) and miR-23-27-24 cluster knockout (KO), *n* = 4 per group. (a) Representative western blots of MBP, PLP, and GAPDH proteins. (b) MBP expression in KO mice was significantly lower than that in WT mice, ^∗^*p* < 0.05. (c) PLP expression in KO mice was significantly lower than that in WT mice.

The expression of MBP and PLP, key proteins involved in the formation of the myelin layer, was analyzed in the spinal cord of 10-week-old mice by western blotting (Figures 6(a)–6(c)). The relative expression of MBP in KO mice (0.19 ± 0.10) was significantly lower than that in wild-type mice (0.86 ± 0.20, *p* = 0.001) (Figure 6(b)). Similarly, the relative expression of PLP in KO mice (0.09 ± 0.05) was also significantly lower than that in wild-type mice (0.24 ± 0.04, *p* = 0.002) (Figure 6(c)). These results suggest that systemic deletion of miR-23-27-24 clusters causes reduced myelination in the CNS.

### 3.4. Oligodendrocyte Differentiation and Myelination in the miR-23 Cluster KO Mice

**Figure 7:**
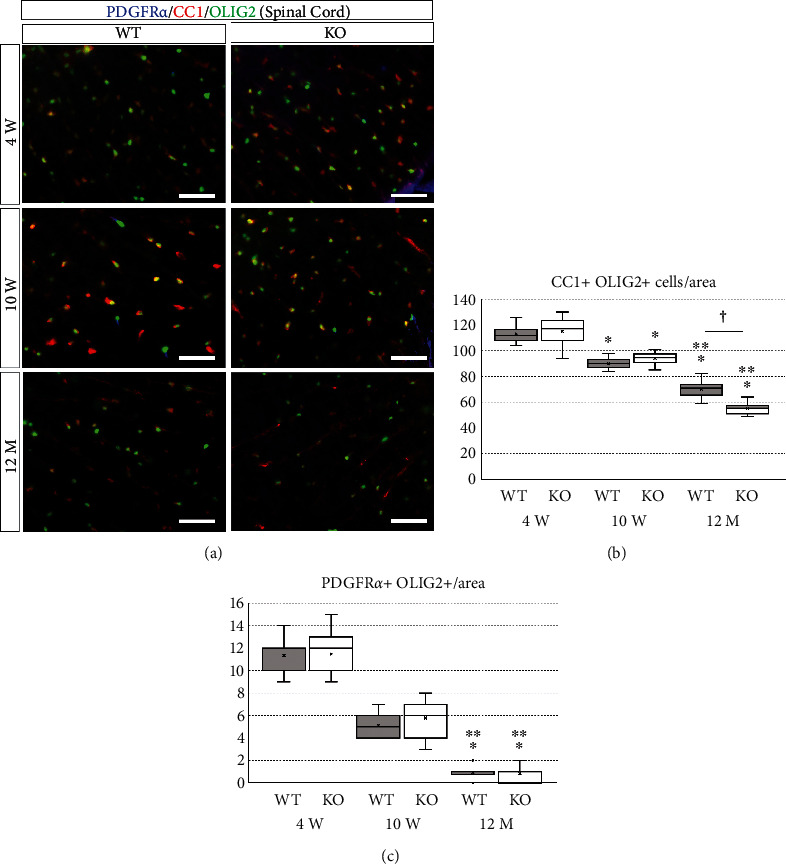
Immunostaining of oligodendrocytes and their progenitor cells in spinal cord sections. Wild-type (WT) and miR-23-27-24 cluster knockout (KO), *n* = 6 per group. (a) Sections of the spinal cord were immunostained for PDGFR*α*, a marker of oligodendrocyte progenitors, CC1, a marker of mature oligodendrocytes, and OLIG2, a broad marker of oligodendrocytes. Scale bars: 50 *μ*m. (b) Number of mature oligodendrocytes showing double positivity for CC1 and OLIG2. The number of mature oligodendrocytes significantly decreased with age in both WT and KO mice. The number of mature oligodendrocytes in KO mice was significantly lower than that in WT mice only at 12 months of age. ^∗^Compared to 4-week-old. ^∗∗^Compared to 10-week-old. ^†^Comparison between WT and KO mice, *p* < 0.05. (c) Number of oligodendrocyte progenitors showing double positivity for PDGFR*α* and OLIG2. The number of oligodendrocyte progenitors at 12 months of age was significantly lower compared to that at 4 and 10 weeks of age in both WT and KO mice. However, there was no significant difference in the number of oligodendrocyte progenitors between WT and KO mice at all ages. ^∗^Compared to 4-week-old. ^∗∗^Compared to 10-week-old, *p* < 0.05.

**Figure 8:**
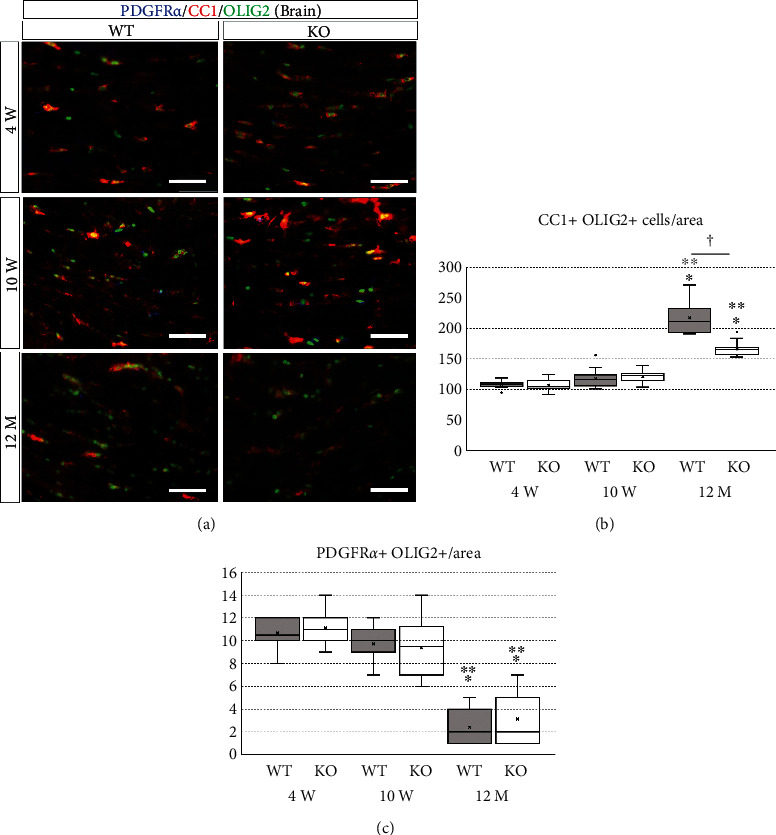
Immunostaining of oligodendrocytes and their progenitor cells in brain sections. Wild-type (WT) and miR-23-27-24 cluster knockout (KO), *n* = 6 per group. (a) Brain sections were immunostained for platelet-derived growth factor receptor *α* (PDGFR*α*), a marker of oligodendrocyte progenitors, CC1, a marker of mature oligodendrocytes, and OLIG2, a broad marker of oligodendrocytes. Scale bars: 50 *μ*m. (b) Number of mature oligodendrocytes showing double positivity for CC1 and OLIG2. The number of mature oligodendrocytes at 12 months of age was significantly lower compared to that at 4 and 10 weeks of age in both WT and KO mice. The number of mature oligodendrocytes in KO mice was significantly lower than that in WT mice only at 12 months of age. ^∗^Compared to 4-week-old. ^∗∗^Compared to 10-week-old. ^†^Comparison between WT and KO mice, *p* < 0.05. (c) Number of oligodendrocyte progenitors showing double positivity for PDGFR*α* and OLIG2. The number of oligodendrocyte progenitors at 12 months of age was significantly lower compared to that at 4 and 10 weeks of age in both WT and KO mice. However, there was no significant difference in the number of oligodendrocyte progenitors between WT and KO mice at all ages. ^∗^Compared to 4-week-old. ^∗∗^Compared to 10-week-old, *p* < 0.05.

To assess the effect of miR-23-27-24 cluster deletion on differentiation and maturation of oligodendrocytes, we performed immunostaining for cell surface markers of OPC and oligodendrocytes in the spinal cord and corpus callosum sections. Mature oligodendrocytes were identified as double positive for OLIG2 and CC1, and OPCs were identified as double positive for OLIG2 and PDGFR*α* (Figures 7 and 8). The number of positive cells per unit area was then measured. In the spinal cord, the number of mature oligodendrocytes was 114.80 ± 9.83 in 4-week-old wild-type mice, 110.93 ± 3.95 in 4-week-old KO mice, 94.133 ± 4.42 in 10-week-old wild-type mice, 89.87 ± 3.85 in 10-week-old KO mice, 53.933 ± 4.27 in 12-month-old wild-type mice, and 70.067 ± 6.41 in 12-month-old KO mice. One-way ANOVA showed significant differences between the groups (*F*(5, 84) = 241.447, *p* < 0.001). The number of mature oligodendrocytes per unit area decreased significantly with age in both wild-type and KO mice (*p* < 0.001) (Figures 7(a) and 7(b)). The number of mature oligodendrocytes in 12-month-old KO mice was significantly lower than that in the age-matched wild-type mice (*p* < 0.001), but there was no significant difference between the KO and wild-type mice at 4 (*p* = 0.467) and 10 weeks (*p* = 0.354) of age (Figures 7(a) and 7(b)). The number of OPCs in the spinal cord was 11.60 ± 1.92 in 4-week-old wild-type mice, 11.00 ± 1.41 in 4-week-old KO mice, 5.80 ± 1.47 in 10-week-old wild-type mice, 5.13 ± 0.99 in 10-week-old KO mice, 0.87 ± 0.64 in 12-month-old wild-type mice, and 0.80 ± 0.56 in 12-month-old KO mice. One-way ANOVA showed significant differences between the groups (*F*(5, 84) = 207.891, *p* < 0.001). The number of OPCs per unit area decreased significantly with age in both wild-type and KO mice (*p* < 0.001), but there was no significant difference between wild-type and KO mice at all ages (*p* = 0.783 in 4-week-old, *p* = 0.699 in 10-week-old, *p* = 1.000 in 12-month-old mice) (Figures 7(a) and 7(c)).

In the corpus callosum, the number of mature oligodendrocytes was 106.53 ± 9.20 in 4-week-old wild-type mice, 107.67 ± 5.77 in 4-week-old KO mice, 120.933 ± 4.42 in 10-week-old wild-type mice, 117.80 ± 14.46 in 10-week-old KO mice, 166.07 ± 12.13 in 12-month-old wild-type mice, and 217.53 ± 26.79 in 12-month-old KO mice. One-way ANOVA showed significant differences between the groups (*F*(5, 84) = 137.177, *p* < 0.001). The number of mature oligodendrocytes in 12-month-old mice was significantly higher than that in 4- and 10-week-old mice in both genotypes (*p* < 0.001) (Figures 8(a) and 8(b)). The number of mature oligodendrocytes in 12-month-old KO mice was significantly lower than that in age-matched wild-type mice (*p* < 0.001), but there was no significant difference between wild-type and KO mice at 4 (*p* = 1.000) and 10 weeks (*p* = 0.992) of age (Figures 8(a) and 8(b)). The number of OPCs in the corpus callosum was 11.00 ± 1.36 in 4-week-old wild-type mice, 10.60 ± 1.12 in 4-week-old KO mice, 9.40 ± 2.53 in 10-week-old wild-type mice, 9.73 ± 1.58 in 10-week-old KO mice, 3.13 ± 2.17 in 12-month-old wild-type mice, and 2.40 ± 1.30 in 12-month-old KO mice. The number of OPCs in 12-month-old mice was significantly lower than that in 4- and 10-week-old mice in both genotypes (*p* < 0.001) (Figures 8(a) and 8(c)). There was no significant difference between wild-type and KO mice at all ages (*p* = 0.989 in 4-week-old, *p* = 0.995 in 10-week-old, *p* = 0.860 in 12-month-old mice) (Figures 8(a) and 8(c)). These results suggest that deletion of the miR-23-27-24 cluster does not inhibit the differentiation of OPCs to mature oligodendrocytes.

**Figure 9:**
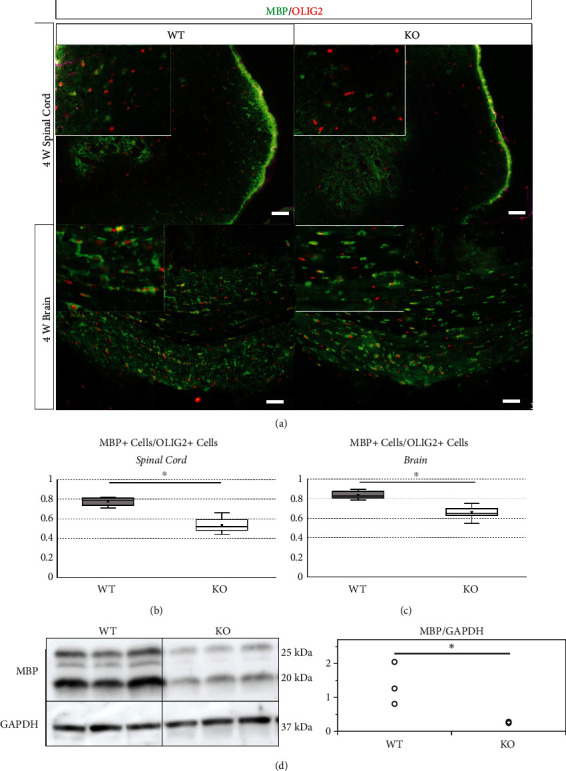
Myelinating oligodendrocytes of the spinal cord and brain at 4 weeks of age. Wild-type (WT) and miR-23-27-24 cluster knockout (KO). (a) Sections of the spinal cord and brain were immunostained for myelin basic protein (MBP) and OLIG2. Cells that were double positive for MBP and OLIG2 were considered myelinating oligodendrocytes. Scale bars: 50 *μ*m. (b) In the spinal cord, the ratio of the number of myelinating oligodendrocytes to the number of OLIG2-positive mature oligodendrocytes was significantly lower in KO mice than that in WT mice, *n* = 6 per genotype, ^∗^*p* < 0.05. (c) In the brain, the ratio of the number of myelinating oligodendrocytes to the number of OLIG2-positive mature oligodendrocytes was significantly lower in KO mice than that in WT mice, *n* = 6 per genotype, ^∗^*p* < 0.05. (d) Representative western blots of MBP and GAPDH proteins in the spinal cord of 4-week-old mice. MBP expression was significantly lower in KO mice than that in WT mice, *n* = 3 per genotype, ^∗^*p* < 0.05.

To assess myelin layer formation by oligodendrocytes, the spinal cord (white matter) and brain (corpus callosum) sections of 4-week-old mice were immunostained for MBP and OLIG2 (Figure 9(a)). The ratio of MBP and OLIG2 double-positive cells to total OLIG2 positive cells was calculated. In the spinal cord, the ratio of double-positive cells in KO mice (0.53 ± 0.07) was significantly lower than that in wild-type mice (0.78 ± 0.04, *p* < 0.001) (Figure 9(b)). Similarly, in the brain, the ratio of double-positive cells in KO mice (0.66 ± 0.05) was significantly lower than that in wild-type mice (0.84 ± 0.04, *p* < 0.001) (Figure 9(c)). In addition, western blotting showed that the relative expression of MBP in the spinal cord of 4-week-old KO mice (0.27 ± 0.02) was significantly lower than that of 4-week-old wild-type mice (1.37 ± 0.63, *p* = 0.038) (Figures 9(d) and 9(e)). These results suggest that deletion of miR-23-27-24 clusters does not affect the differentiation of OPCs to oligodendrocytes but inhibits myelin layer formation by oligodendrocytes.

### 3.5. Targets and Downstream Signaling Pathways Regulated by the miR-23-27-24 Cluster

**Figure 10:**
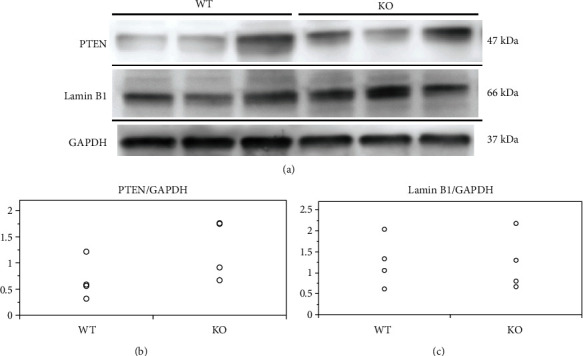
Expression of candidate targets of miR-23-27-24 cluster. Wild-type (WT) and miR-23-27-24 cluster knockout (KO). (a) Representative western blots depicting phosphatase and tensin homolog deleted on chromosome 10 (PTEN) and lamin B1 levels in the spinal cord of 10-week-old mice. (b) PTEN expression was not significantly different between WT and KO mice, *n* = 5 per genotype. (c) Lamin B1 expression was not significantly different between WT and KO mice, *n* = 5 per genotype. (d) Proteome analysis of the spinal cord tissue from 10-week-old mice, *n* = 1 per genotype. Leucine-zipper-like transcription regulator 1 (LZTR1) was strongly expressed in KO mice compared to WT mice.

PTEN and lamin B1, which are involved in regulating myelination, are the known targets of miR-23 [9, 12]. We evaluated the expression of PTEN and lamin B1 in the white matter of the spinal cord of 10-week-old mice by western blotting (Figure 10(a)). Results showed no significant differences in the relative expression of PTEN (wild-type, 0.67 ± 0.33; KO, 1.27 ± 0.56; *p* = 0.087) and lamin B1 (wild-type, 1.48 ± 0.72; KO, 1.24 ± 0.68; *p* = 0.618) between wild-type and KO mice (Figures 10(b) and 10(c)).

**Figure 11:**
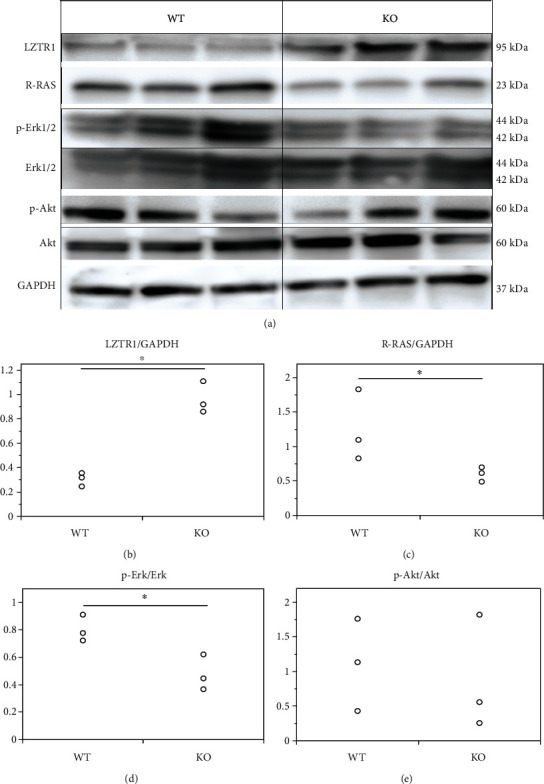
Expression of LZTR1 and its downstream signaling factors. Wild-type (WT) and miR-23-27-24 cluster knockout (KO), *n* = 3 per genotype. (a) Representative western blots of LZTR1, R-RAS, Erk1/2, phospho-Erk1/2 (p-Erk1/2), Akt, phospho-Akt1/2 (p-Akt), and GAPDH. (b) The expression of LZTR1 was significantly higher in KO mice than in WT mice, ^∗^*p* < 0.05. (c) The expression of R-RAS was significantly lower in KO mice than in WT mice, ^∗^*p* < 0.05. (d) The ratio of p-Erk1/2 expression to Erk1/2 expression was significantly lower in KO mice than in WT mice, ^∗^*p* < 0.05. (e) The ratio of p-Akt expression to Akt expression was not significantly different between KO and WT mice.

DIA proteome analysis was performed to identify novel targets of the miR-23-27-24 cluster using the spinal cord white matter from 10-week-old mice. Among the candidate target genes of the miR-23-27-24 cluster predicted using the TargetScan database, LZTR1 was found to be strongly upregulated upon deletion of the miR-23-27-24 cluster. LZTR1 has been reported to regulate RAS signaling [25] [26]. Among several types of RAS, R-RAS plays an essential role in regulating myelination by synergistically activating Erk1/2-MAPK and PI3K/Akt signaling pathways [27]. We evaluated the expression of LZTR1, R-RAS, p-Erk1/2, Erk1/2, p-Akt, and Akt in the white matter of the spinal cord of 10-week-old mice by western blotting (Figure 11). Results showed that the relative expression of LZTR1 in KO mice (0.96 ± 0.13) was significantly higher than that in wild-type mice (0.32 ± 0.06, *p* = 0.002) (Figures 11(a) and 11(b)). Conversely, the relative expression of R-RAS in KO mice (0.60 ± 0.10) was significantly lower than that in wild-type mice (1.25 ± 0.52, *p* = 0.043) (Figures 11(a) and 11(c)). The ratio of p-Erk1/2 (active form of Erk1/2) expression to Erk1/2 expression was significantly lower in KO mice (0.48 ± 0.13) than in wild-type mice (0.80 ± 0.10, *p* = 0.025) (Figures 11(a) and 11(d)). In contrast, there was no obvious difference in the ratio of p-Akt (active form of Akt) expression to Akt expression between wild-type mice (1.11 ± 0.67) and KO mice (0.88 ± 0.83, *p* = 0.726) (Figures 11(a) and 11(e)). Thus, deletion of the miR-23-27-24 cluster increased the expression of LZTR1, decreased the expression of R-RAS, and suppressed the activation of Erk1/2 but had no effect on Akt activation.

## 4. Discussion

Our results demonstrated that systemic deletion of the miR-23-27-24 cluster reduced myelination in the white matter of the brain and spinal cord, resulting in impaired motor function. Further, deletion of the miR-23-27-24 cluster did not affect the differentiation of OPCs to oligodendrocytes but inhibited myelin layer formation by oligodendrocytes. In addition, deletion of the miR-23-27-24 cluster increased LZTR1 expression, decreased R-RAS expression, and suppressed Erk1/2 activation.

Previous studies showed that the miR-23-27-24 cluster is expressed in the lung, skeletal muscle, and liver [18]. Our recent previous study demonstrated that the miR-23-27-24 cluster is expressed in these organs as well as in the CNS [28]. Since the miR-23-27-24 cluster is expressed in skeletal muscles, it could be argued that motor impairment upon miR-23-27-24 cluster deletion is possibly due to the loss of skeletal muscle function. However, in a previous study, deletion of the miR-23-27-24 cluster did not induce functional changes in skeletal muscles [18]. Therefore, the decline in motor function in our study might be due to CNS abnormalities. In the present study, the knockout mice showed no significant motor deficits and no abnormalities in the open field test or the balance beam test. However, only the hanging wire test showed inferior results in the knockout mice compared to wild-type mice. In the miR-23-27-24 cluster KO mice, the myelin sheath was thinner, but the myelin sheath was not completely lost; therefore, motor function may not have been substantially impaired. In previous studies using mouse models with hypomyelination, motor function has been assessed by several specialized devices to detect minor abnormalities, rather than by the usual gait analysis [29, 30]. The hanging wire test is one such device, and in the present study, it allowed us to show significant differences between wild-type mice and KO mice. Abnormalities in myelination in the central nervous system may have led to abnormalities in balance and movement coordination when clinging to the wire.

In KO mice, hypoplasia of the white matter was observed at 10 weeks and 12 months of age, and hypomyelination was observed at all ages (4 weeks, 10 weeks, and 12 months of age). Previous studies have shown that normal oligodendrocyte differentiation is essential for normal myelination [31–34]. During oligodendrocytes differentiation, OPCs differentiate into premyelinating oligodendrocytes, which further differentiate into myelinating oligodendrocytes, cells that initiate myelination [3]. We observed that deletion of the miR-23-27-24 cluster had no effect on the number of mature oligodendrocytes during the early growth period (4 and 10 weeks) and on the number of OPCs at all ages. These results suggest that reduced myelination in the miR-23-27-24 cluster KO mice is not due to the inhibition of oligodendrocyte differentiation. The expression of MBP, which plays an important role in the process of myelination [35, 36], was decreased in 4-week-old KO mice. In addition, the expression of PLP, which plays an important role in the formation and maintenance of the myelin multilayer structure [37, 38], was also decreased in 10-week-old KO mice. These findings suggest that the miR-23-27-24 cluster is involved in regulating later stages of differentiation of OPCs to myelinating oligodendrocytes.

It has been previously reported that some miRNAs in the miR-23-27-24 cluster are independently involved in regulating myelination [9, 12, 14, 15]. A previous study showed that lamin B1 overexpression suppressed the expression of MBP and PLP, and lamin B1 expression was negatively regulated by miR-23 [9]. Another study showed that the expression of MBP and PLP was upregulated and myelination was increased in the transgenic mice overexpressing miR-23a in oligodendrocytes. Furthermore, the transgenic mice showed decreased expression of PTEN and activation of the Akt signaling [12]. Thus, miR-23 functions as a positive regulator of myelination. However, miR-27a has also been identified as a negative regulator of myelination. A previous study showed that overexpression of miR-27a inhibited the differentiation of OPCs to mature oligodendrocytes via regulation of the Wnt/*β*-catenin signaling pathway. In the present study, miR-23 and miR-27a were deleted simultaneously. In the KO mice, MBP and PLP expressions and myelin formation were reduced, but contrary to previous studies, oligodendrocyte differentiation was not inhibited, and lamin B1 and PTEN expressions were not significantly increased. The contradiction between the previous and our results may be the difference between the in vitro model and the in vivo model. Differences in the expression patterns of individual microRNAs in the central nervous system (Figure 1) may also have affected the results. Furthermore, since miRNAs in a cluster work in a complementary manner [13], deletion of miRNA clusters may have different effects than those due to deletion of individual miRNAs. We should further examine the processing mechanisms to individual mature miRNA of miR-23-27-24 cluster.

In this study, LZTR1 was identified as a novel target of the miR-23-27-24 cluster. In the TargetScan database, LZTR1 is a target of miR-23b and miR-27a. LZTR1 is known to regulate RAS signaling [25]. Among various types of RAS, R-RAS has been reported to play essential roles in regulating myelination by synergistically activating PI3K/Akt/mechanistic target of rapamycin (mTOR) and Mek/Erk1/2-MAPK signaling pathways [27, 29]. Herein, deletion of the miR-23-27-24 cluster upregulated the expression of LZTR1 and downregulated the expression of R-RAS. Furthermore, deletion of the miR-23-27-24 cluster did not affect the activity of the PI3K/Akt/mTOR pathway but reduced the activity of the Mek/Erk1/2-MAPK pathway. A recent study reported that Mek/Erk1/2-MAPK signaling and PI3K/Akt/mTOR signaling pathways are cooperatively involved in regulating myelination, but their cooperativity is altered at different stages of myelination [39]. A previous study showed that although oligodendrocyte differentiation was suppressed in mTOR knockout mice, it remained suppressed even after conditional overactivation of ERK1/2 [39]. In addition, Erk1/2 knockout mice showed no change in the number of differentiated oligodendrocytes during the first 2 weeks of age, when oligodendrocyte progenitors rapidly differentiate in the spinal cord [40]. Therefore, the PI3K/Akt/mTOR pathway, and not the Mek/ERK1/2-MAPK pathway, is the key regulator of oligodendrocyte differentiation and initiation of myelination. In contrast, it has been reported that myelin growth is promoted in adults by forced overactivation of the Mek/ERK1/2-MAPK and PI3K/Akt/mTOR pathways [41–43]. However, while strong ERK1/2 signaling persists in the adult spinal cord, mTOR expression in oligodendrocytes declines to almost undetectable levels during adulthood [44]. Additionally, tamoxifen-induced elimination of ERK1/2 from mature oligodendrocytes in young adults resulted in significant downregulation of myelin gene expression and formation of abnormal myelin structures, whereas elimination of mTOR from adult oligodendrocytes in a similar manner produced no obvious myelin abnormalities [44]. Therefore, the Mek/Erk1/2-MAPK pathway, and not the PI3K/Akt/mTOR pathway, may be predominantly involved in myelin growth in adults. In the present study, deletion of the miR-23-27-24 cluster suppressed the activity of only the Mek/Erk1/2-MAPK pathway independently of the PI3K/Akt/mTOR pathway, which may have inhibited myelination without affecting oligodendrocyte differentiation.

There were some limitations to the present study. Namely, the deletion of the miR-23-27-24 cluster was not specific to myelin but systemic, so we could not exclude effects from other organs. In addition, because we did not use inducible knockout mice in this study, we were not able to identify the critical timing at which the miR-23-27-24 cluster exerts its function. Future studies using inducible knockout mice of the myelin-specific miR-23-27-24 cluster are needed.

**Figure 12:**
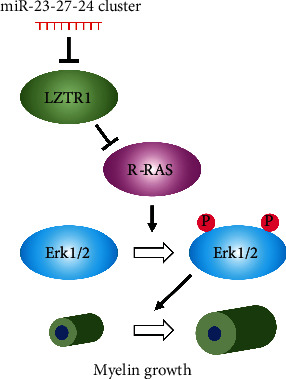
Role of miR-23-27-24 clusters in myelin formation. The miR-23-27-24 cluster enhances myelin growth in adults by negatively regulating LZTR1, promoting R-RAS expression, and activating the Mek/Erk1/2-MAPK pathway.

However, based on the results of the present study, we conclude that the miR-23-27-24 cluster activates the Mek/Erk1/2-MAPK pathway by negatively regulating LZTR1 and promoting R-RAS expression, thereby promoting myelin sheath formation by mature oligodendrocytes (Figure 12).

## Figures and Tables

**Table 1:** Expression of miR-23-27-24 cluster in the central nervous system.

	Spinal cord			Brain		
	WT	KO	*p* value	WT	KO	*p* value
miR-23a	1.00 ± 0.31	0.02 ± 0.01	<0.001	1.00 ± 0.24	0.00 ± 0.00	<0.001
miR-23b	1.00 ± 0.26	0.00 ± 0.00	<0.001	1.00 ± 0.11	0.00 ± 0.00	<0.001
miR-27a	1.00 ± 0.14	0.01 ± 0.00	<0.001	1.00 ± 0.28	0.01 ± 0.00	<0.001
miR-27b	1.00 ± 0.25	0.00 ± 0.00	<0.001	1.00 ± 0.11	0.00 ± 0.00	<0.001
miR-24	1.00 ± 0.25	0.00 ± 0.00	<0.001	1.00 ± 0.29	0.00 ± 0.00	<0.001

Mean values ± SD of relative expressions to the mean expressions in wild-type mice. Wild-type (WT) mice and miR-23-27-24 cluster knockout (KO) mice. Statistical differences between WT mice and KO mice were calculated.

**Table 2:** Expression of miR-23-27-24 cluster in spinal cord at 4 weeks, 10 weeks, and 12 months old.

		4 W	10 W	12 M
WT	miR-23a	1.11 ± 0.25	5.13 ± 1.00	2.77 ± 1.17
	miR-23b	0.95 ± 0.38	13.68 ± 8.67	18.32 ± 13.27
	miR-27a	0.82 ± 0.30	15.03 ± 3.41	10.35 ± 6.11
	miR-27b	1.10 ± 0.59	13.11 ± 1.78	9.15 ± 5.42
	miR-24	0.93 ± 0.24	11.33 ± 2.32	8.23 ± 4.33

KO	miR-23a	0.02 ± 0.01	0.03 ± 0.01	0.01 ± 0.01
	miR-23b	0.00 ± 0.00	0.01 ± 0.01	0.00 ± 0.00
	miR-27a	0.02 ± 0.01	0.01 ± 0.01	0.01 ± 0.02
	miR-27b	0.00 ± 0.00	0.00 ± 0.00	0.00 ± 0.00
	miR-24	0.00 ± 0.00	0.01 ± 0.00	0.00 ± 0.00

Mean values ± SD of relative expressions to the expressions in a 4-week-old wild-type mouse. Wild-type (WT) mice and miR-23-27-24 cluster knockout (KO) mice.

**Table 3:** Data from behavior tests.

		WT	KO	*p* value
Total distance (cm)	4 W	1129.11 ± 147.87	1064.80 ± 189.60	0.998
	10 W	1661.96 ± 245.66	1530.59 ± 181.81	0.823
	12 M	1553.40 ± 220.62	1564.81 ± 316.81	1.000

Duration to fall (sec)	4 W	46.43 ± 19.51	40.39 ± 10.30	0.981
	10 W	146.21 ± 19.12	48.83 ± 22.33	<0.001
	12 M	13.62 ± 3.88	4.43 ± 2.32	0.877

Duration to reach (sec)	4 W	4.25 ± 0.37	4.48 ± 0.46	1.000
	10 W	3.53 ± 0.26	3.66 ± 0.34	1.000
	12 M	10.81 ± 3.34	12.48 ± 1.81	0.361

Mean values ± SD. Wild-type (WT) mice and miR-23-27-24 cluster knockout (KO) mice. Statistical differences between WT mice and KO mice were calculated.

## Data Availability

The data that support the findings of this study are available from the corresponding author upon reasonable request.
